# 3D-Printed Ergonomically Designed Feeding Aid for Patients With Limited Manual Dexterity

**DOI:** 10.7759/cureus.37089

**Published:** 2023-04-04

**Authors:** Shreya Colvenkar, Aditya Mohan Alwala, Tejaswini PSS, Sneha Bharadwaj, MD Shakeel Ahmed

**Affiliations:** 1 Department of Prosthodontics, Manthena Narayana Raju (MNR) Dental College and Hospital, Sangareddy, IND; 2 Department of Oral and Maxillofacial Surgery, Manthena Narayana Raju (MNR) Dental College and Hospital, Sangareddy, IND; 3 Department of Oral and Maxillofacial Surgery, Malla Reddy Institute of Dental Sciences, Hyderabad, IND; 4 Department of Prosthodontics, Malla Reddy Dental College for Women, Hyderabad, IND

**Keywords:** dexterity, 3d printed, handle, feeding, customized

## Abstract

Self-eating is a difficult task when hand and finger movements are restricted. Many patients find difficulty in holding utensils because of slender handles. It is essential to have a customized handle that will allow a better grip on traditional feeding aid. When the handle is customized to the patient’s hands and fingers, patients will have better control while eating. This reduces their dependency on others, thus helping to regain their lost self-esteem. This technical report describes a simple method of fabricating a three-dimensional (3D)-printed ergonomically designed handle for a feeding aid from materials readily available in the dental office.

## Introduction

The ability to use the hand efficiently for functional tasks such as eating, writing, and even brushing needs a good range of motion in the fingers. At a young age, dexterity is good, but with aging, it becomes diminished. Patients with neuromuscular degenerative conditions present with reduced grip strength. Limited dexterity is commonly seen in arthritis, Parkinson’s, congenital abnormality, and trauma patients [1-4]. Patients with limited dexterity find difficulty in self-feeding. Many patients find difficulty in holding the standard slender design of utensils such as cups, spoons, and forks because of their diminished ability to grip thinner objects. Adaptive feeding aids are well-recognized means for self-feeding in elderly patients or patients with disability [5-12]. Self-feeding is very important to regain control while eating meals. This in turn will increase their self-confidence due to less dependency on caregivers [9]. Arthritis patients suffer from stiffness, limited motion of the hand and fingers, and joint pain. If these patients have the determination to eat, then customized feeding aid will be a helpful aid. The customized feeding utensil should have a handle that is easy to grip and fits correctly in one’s hand to carry the food in the mouth.

This technical report describes a simple technique to customize feeding aid with three-dimensional (3D)-printed handle for patients with limited dexterity. The handle customized to patient’s hands and fingers can help in self-feeding.

## Technical report

**Figure 1:**
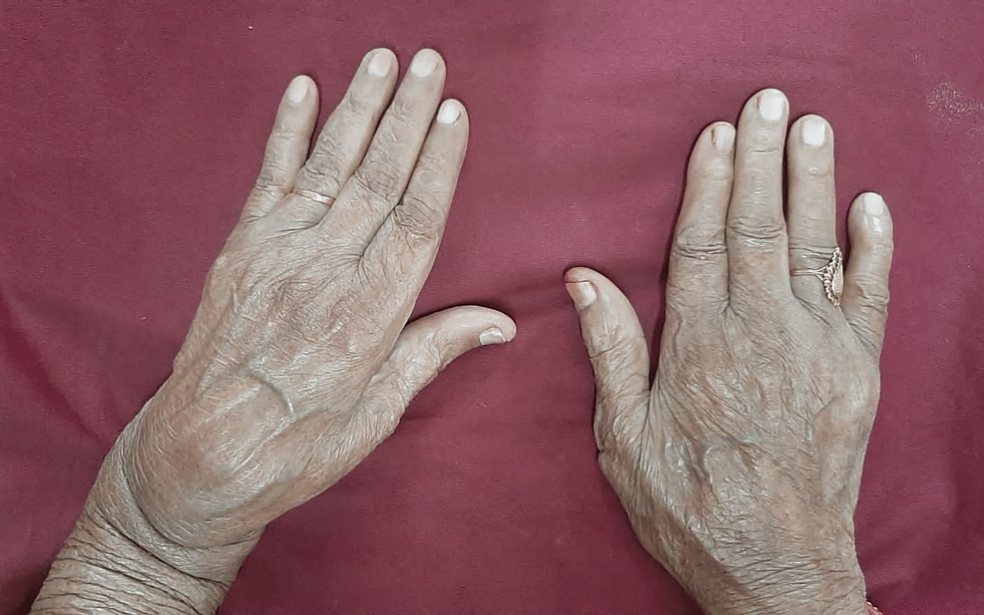
Swelling in the left hand

Make the patient comfortable and calm by genuinely acknowledging the concerns about treatment. Explain step-by-step the procedure to be carried out. Select the feeding aid of the patient’s choice, and grease it with a separating medium. Take a putty impression material, and mix according to the manufacturer’s instructions. Once soft, gently mold it around the handle of the cup. Request the patient to gently squeeze the material, such that it is comfortable to hold and drink water (Figure 1).

**Figure 2:**
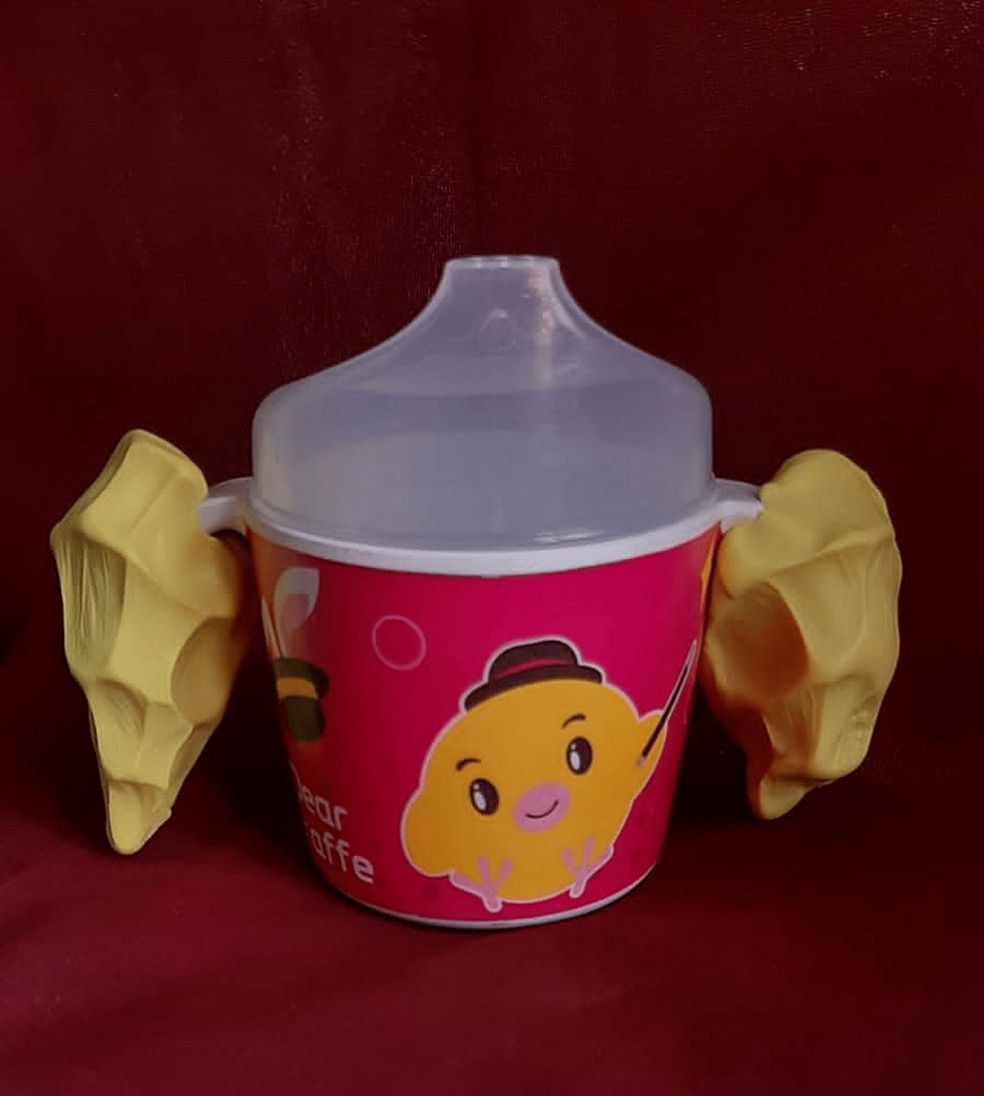
Molded handle for feeding cup

Once polymerization is complete, separate it from the cup (Figure 2).

**Figure 3:**
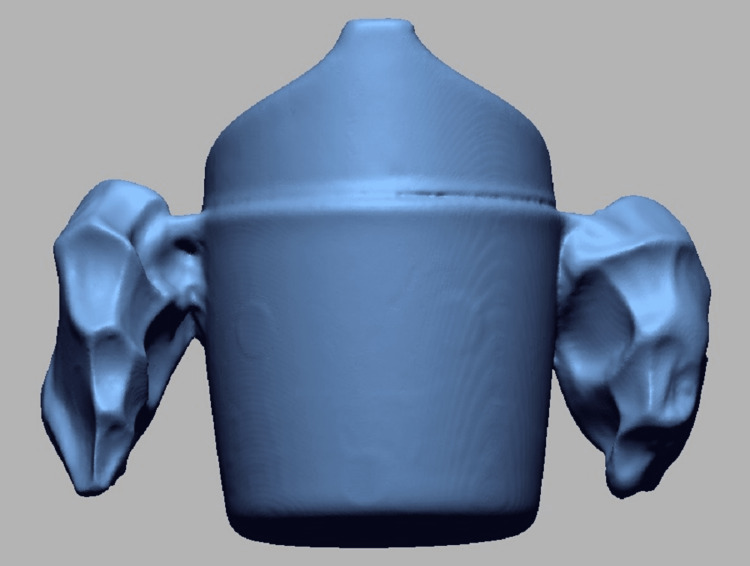
Scanning

Manufacture the handle using the 3D-printed software Mimics (Materialise NV, Leuven, Belgium). Scan the mold and the handle separately. Scan the surface of the mold and the handle. The computer-aided design (CAD) file of the handle was inserted into the CAD file of the mold, and Boolean operation was performed so that there is a hollowness created in the mold. This facilitated easy penetration and fit of the handle (Figure 3).

**Figure 4:**
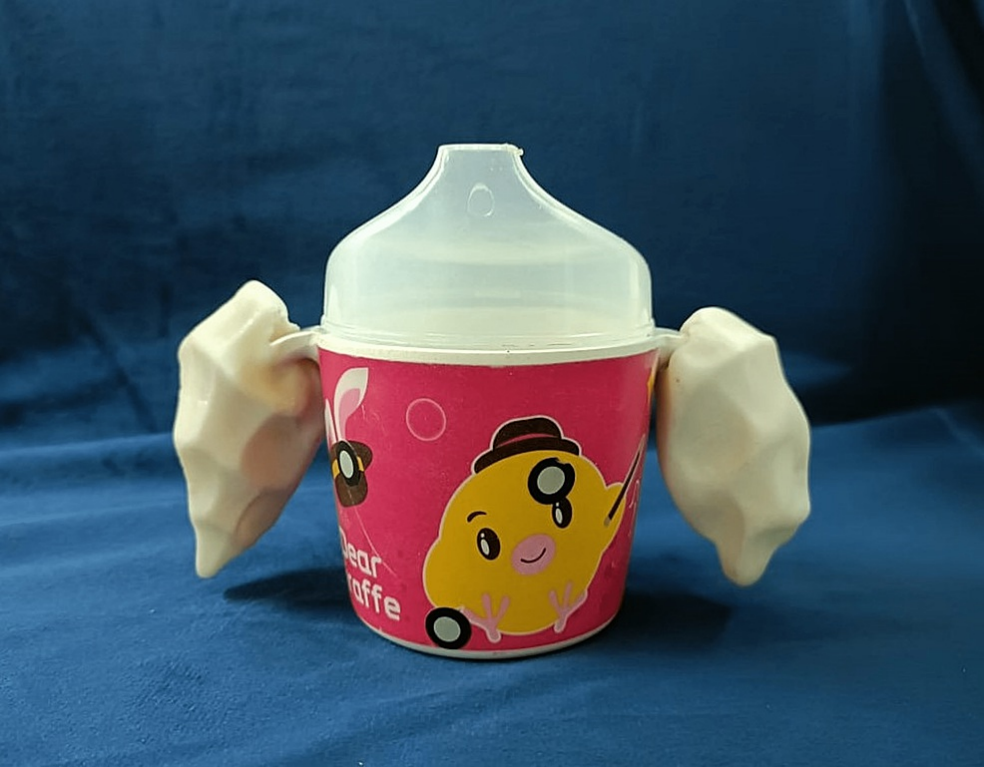
3D-printed handle

Print the handle with the 3D printer Ultimaker 2+ (Ultimaker BV, Framingham, MA) using a polylactic acid material (Figure 4). Minor adjustments were made to correctly insert the handle into the cup.

**Figure 5:**
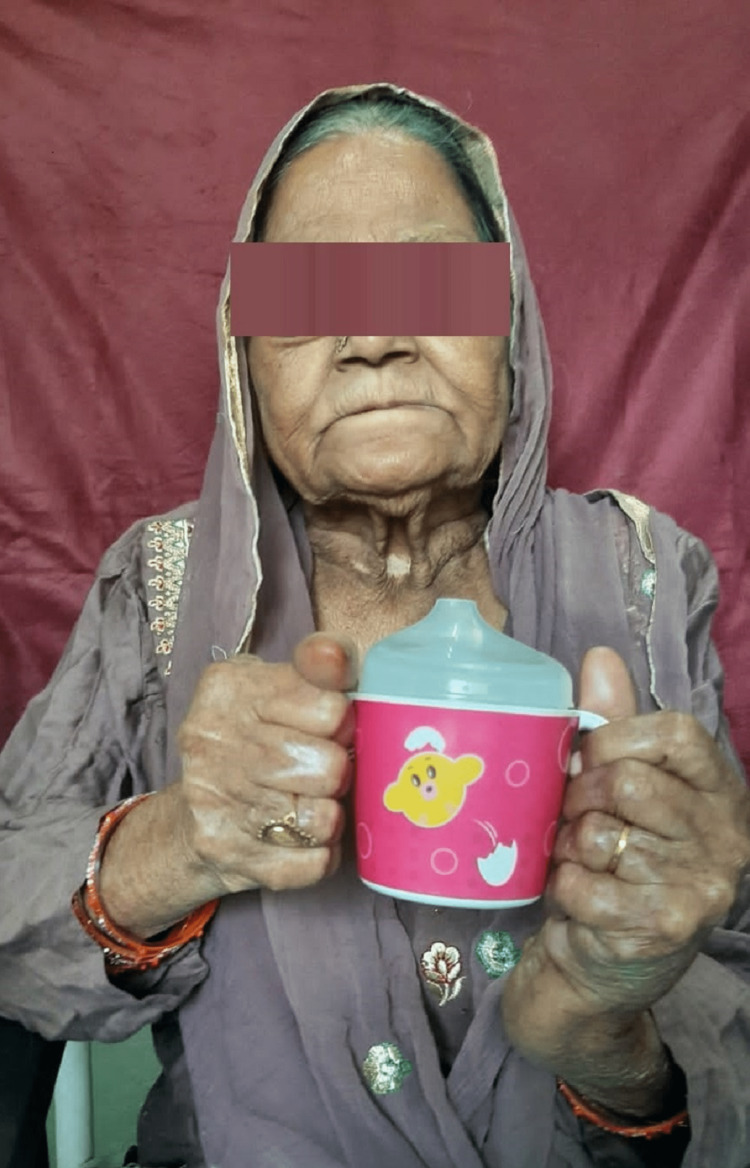
The patient holding the cup with customized handle

Instruct the patient about the handling of the feeding aid (Figure 5).

## Discussion

It is crucial for some individuals to maintain their independence forever. Performing simple daily activities brings confidence and pride in them, no matter how small and insignificant it might seem to others. Eating is one of the examples of the most important activities in daily life. But for patients with limited manual dexterity, it becomes a chore. A literature search shows that life expectancy is better in individuals who maintain their independence. In his study, Gustafsson concluded that disabled individuals who attained their goal of self-nourishment had an increased sense of control, hope, and security for the future [13].

Patients with limited dexterity find difficulty in holding slim, slender handles of conventional cups and other utensils. Brach and his colleagues emphasized that conventional cutlery requires manual gripping with a greater range of motion [14]. Few adaptive aids have been mentioned in the literature, but they are not customized to individual patients [5-12]. A study conducted by van Roon and Steenbergen has shown that patients with cerebral palsy who used thickened spoon handle were faster in picking up the spoon and transporting it to the bowl, especially when the spoon was filled with water without increased spilling [8].

McDonald and his colleagues concluded that participants handling cutlery with small and medium thickeners were able to perform the task of reaching food in a bowl arranged on a table with shorter movement lengths [8].

Adjusting the design of feeding aid, for patients with limited manual dexterity, is predominantly based on the specialist notion of occupational therapist and physiotherapist. A literature search mentions cutlery with large-diameter handles to assist in feeding, but none of them are customized to the patient’s fingers and hands. This technical report mentions the use of a putty impression material, which is easily available in the dental office to mold the handle of the feeding aid. It is then printed with polylactic acid material. 3D printing has gained popularity because of its accuracy, reduced chair time, and accelerated production time. In 3D printing, three-dimensional objects are created with the help of computer software. Polylactic acid material has good dimensional stability, durability, strength, and finish. Colvenkar et al. described a technique for the fabrication of 3D-printed toothbrush holder for patients with limited manual dexterity [15].

In the present case, the patient was not able to hold the cup with water in her right hand. Also, due to the slender handle, the patient could not hold the cup with both hands due to the limited dexterity in the left hand. With a customized handle, the patient could easily drink water, giving her a feeling of self-independence. During the one-month follow-up, the patient did not have any complaints. The use of an individually modeled 3D-printed handle made drinking a regular activity rather than a task. The handle could be easily removed and reinserted in the cutlery whenever needed. Since its water-resistant, it was easy to clean.

## Conclusions

This article describes an easy method for making 3D-printed handles for feeding aid for patients with limited manual dexterity with materials easily available in dental setups. A large-diameter handle allowed a better grip on the cup, thus making the patient more independent while feeding. Material manipulation can be made chairside by a dentist and then 3D-printed with polylactic acid material.

## References

[1] Gonsalves WC, Wrightson AS, Henry RG (2008). Common oral conditions in older persons. Am Fam Physician.

[2] Fiest KM, Hitchon CA, Bernstein CN (2017). Systematic review and meta-analysis of interventions for depression and anxiety in persons with rheumatoid arthritis. J Clin Rheumatol.

[3] VanDyke MM, Parker JC, Smarr KL, Hewett JE, Johnson GE, Slaughter JR, Walker SE (2004). Anxiety in rheumatoid arthritis. Arthritis Rheum.

[4] Opara J, Brola W, Leonardi M, Błaszczyk B (2012). Quality of life in Parkinson’s disease. J Med Life.

[5] Ma HI, Hwang WJ, Chen-Sea MJ, Sheu CF (2008). Handle size as a task constraint in spoon-use movement in patients with Parkinson's disease. Clin Rehabil.

[6] Sabari J, Stefanov DG, Chan J, Goed L, Starr J (2019). Adapted feeding utensils for people with Parkinson’s-related or essential tremor. Am J Occup Ther.

[7] Connolly MJ, Wilson AS (1990). Feeding aids. BMJ.

[8] McDonald SS, Levine D, Richards J, Aguilar L (2016). Effectiveness of adaptive silverware on range of motion of the hand. PeerJ.

[9] Shinnar SE (1983). Use of adaptive equipment in feeding the elderly. J Am Diet Assoc.

[10] Nelson SE (1983). Counterbalanced swivel fork. Am J Occup Ther.

[11] Mills M (1983). A gooseneck feeding device. Am J Occup Ther.

[12] Shaw G, Wright C (1982). A two-handle spoon: an aid for independent eating. Am J Occup Ther.

[13] Gustafsson B (1995). The experiential meaning of eating, handicap, adaptedness, and confirmation in living with esophageal dysphagia. Dysphagia.

[14] Brach JS, VanSwearingen JM, Newman AB, Kriska AM (2002). Identifying early decline of physical function in community-dwelling older women: performance-based and self-report measures. Phys Ther.

[15] Colvenkar S, Kunusoth R, Prakash R, Alwala AM, Ashok Kumar S (2022). Individually modeled 3D printed toothbrush and interproximal brush handle with name for patients with limited manual dexterity. Cureus.

